# Guarantee of Interneships

**Published:** 1913-04

**Authors:** 


### BUFFALO MEDICAL JOURNAL

**A Monthly Review of Medicine and Surgery**

EDITOR AND PUBLISHER DR. A. L. BENEDICT, 228 Summer St., corner of Elmwood Ave., Buffalo. (Address for all communications. Please make personal and telephone calls before 1 P. M.)

THE BUFFALO MEDICAL JOURNAL is the third, in age, in the western continent. It is independent but supports professional organization. As a scientific medium, it is unrestricted in territory. As a news medium, it is limited mainly to the western four districts of the Medical Society of the State of New York. The co-operation of physicians is asked in securing personal, obituary and other news items. Secretaries of Medical Societies in this region are requested to send brief reports of meetings. Full ransactions will be published at cost of composition.

Subscription $2.00 a year in advance; $3.00 for two years in advance. Changes and errors in address or failure or duplication of delivery, should be reported immediately, Back numbers will be supplied if possible but requests for copies should be made promptly,

YEARLY VOLUME 68. APRIL, 1913 NO. 9

*The injunction, "Eat what you like," is justified only as it implies "Eat what you lack."*

---

### Guarantee of Interneships.

The recent decision to increase the number of years of medical study to five in Pennsylvania, the last year consisting mainly of hospital work, and the gradual insistance on experience as interne by various government services and civil service boards, establishes a precedent which must result in a guarantee of such practical experience by every medical college in the country.

While it will probably be several years before hosptial service will be an actual requirement for license by state boards generally, and while practical training in hospitals does not necessarily imply an interneship or conflict with the continuance of the institution of interne service under the control of the hospital itself, it is none too soon for the medical profession to discuss seriously the problem involved.

A quarter of a century ago there were comparatively few hospitals; those that existed were almost entirely confined to large cities, and, except for the very large cities, these were almost entirely of the general type and of relatively large size. At present there are few towns of 10,000 inhabitants without a hospital, and there many villages well provided in this respect. Private or semi-private hospitals have multiplied and there are many hospitals more or less specialized in their field of work.

On the one hand, the large, general hospitals scarcely afford an adequate supply of interneships; on the other hand, the small and special hospitals can scarcely find enough graduates to perform the duties of internes. The employment of undergraduates as internes interferes with the college work, and, conversely, the demands of class attendance interfere with the proper performance of duties by the internes.

The problem of fulfilling the legal or economic requirement of a fifth, hospital year, can be met by the extension of the system of ward and laboratory classes and, in many respects, proper regulation of instruction can be best secured by this plan. This method would not directly interfere with the present custom of interne service. But, certain objections may be foreseen. In the first place, it is altogether likely that the average graduate, whose investment of time in preliminary and technical training will bring him to a minimum age of twenty-five instead of twenty or twenty-one, will not be anxious to accept further interne service unless at a salary (the old method of no definite preliminary training and two or three years of medical attendance having made it perfectly possible and by no means rare for a bright boy to graduate at twenty, and have his diploma withheld till he should have reached his majority, meanwhile serving as interne). This objection is not without its bright side. In the second place, a formal appointment as interne is superior to any possible system of class work by affording opportunities for emergent experience and by adding to the technical training, the maturing influence of professional and institutional responsibility. Thirdly, the undue extension of the system of ward classes involves an appreciable risk to patients, not present, except by gross indiscretion, under the interne system. Fourthly, the presence of students, who have finished their course, except for practical work, and who have the responsibility only of students, can scarcely fail to magnify the friction due to officiousness, greater personal acquaintance with patients, ambition, etc., already noticed to some degree. There will hence, be a tendency, both on the part of attendants, internes and patients and of the individuals and organizations supporting benevolent institutions, to favor hospitals not used for teaching.

How far these theoretic objections will manifest themselves practically can only be conjectured, and it is possible that they may be mere apprehensions. At any rate, it should be clearly understood that they are not presented here as either original or personal convictions of the editor.

If the validity of these objections is not recognized, the fifth year of medical study will develop as an extension of ward and laboratory classes, with special lectures and demonstrations and whatever additional problems are created regarding hospital management and supply of internes will be met as they arise, experience gradually indicating the best solutions. It is altogether likely that there will also arise a protest from students, particularly those contemplating some specialized practice, certain accepted forms of post graduate study, or having opportunities for something like the old system of preceptorship.

It is not so much the personal conviction of the validity of these disadvantages as the fact that, already, most well equipped colleges can place 90 per cent of their graduates as internes and a personal appreciation of the value to the individual student of a formal interneship, as well as of the value of his services to the average hospital, that inclines us to favor the other method of solution. This consists in the adaptation of interne service to the requirements of laws regarding graduation and license. But this method of solving the problem also involves some difficult corollaries.

First of all, every medical college will be obliged to guarantee promptly, upon the completion of the didactic course, an adequate interne service. To do so, it must control, not only informally but by legally invested power, a large amount of clinical material in hospitals. To secure this power, without resort to arbitrary methods at variance with ethical ideals and legal precedent for this country, both the college and hospital must be under more formal legal control and must be more generally representative of the medical profession. On the whole, these prerequisites are rather favorable to professional solidarity and efficiency than otherwise and while involving some personal sacrifices, they will be beneficial.

Secondly, the plan of elevating interne service to a part of the medical curriculum will require some systematic attempt to solve the problem of service in small and specialized hospitals. A similar problem is already realized in regard to nursing but has not been met. Both problems can be solved by exchange of services so as to afford a proper generalization of experience for both physician and nurse in training. This solution implies a waiving of certain personal preferences, a compulsory co-operation among hospitals not strictly private and various minor inconveniences.

The ultimate solution of all the details of the problem will, of course, require years, and it cannot be accurately forseen how all the necessary arrangements will be perfected. We have not attempted to prophesy nor to present personal views beyond the degree inevitable to anyone who essays a consideration of a novel problem and who must, therefore, trace some imaginary routes in an unchartered territory and point out the probable advantages and disadvantages of each. What we want to emphasize is that the time at which the solution will be required is inevitably approaching, perhaps rapidly, for we live in an age of swift changes; and that fewer serious errors will be made if the profession seriously considers the problem in advance of an emergency, and acts concertedly.

